# First report of peritonitis caused by the vancomycin-resistant coccus *Pediococcus pentosaceus* in a patient on continuous ambulatory peritoneal dialysis

**DOI:** 10.1099/acmi.0.000007

**Published:** 2019-04-23

**Authors:** Shefali Gupta, Chinmoy Sahu, Soumyabrata Nag, Uma Shankar Saha, Narayan Prasad, Kashi Nath Prasad

**Affiliations:** 1 Department of Microbiology, Sanjay Gandhi Postgraduate Institute of Medical Sciences, Lucknow, India; 2 Department of Nephrology, Sanjay Gandhi Postgraduate Institute of Medical Sciences, Lucknow, India

**Keywords:** peritoneal dialysis, *Pediococcus pentosaceus*, fever, ciprofloxacin

## Abstract

**Introduction:**

Worldwide, about one-tenth of end-stage renal disease (ESRD) patients are on peritoneal dialysis (PD). Peritonitis is a major cause of PD failure and change of therapy to haemodialysis. An update on peritoneal dialysis-related infections has recommended the use of a first generation cephalosporin or vancomycin as an empirical therapy for Gram-positive organisms. *Pediococcus* spp. is a Gram-positive environmental cocci that have been increasingly reported from various nosocomial infections but very rarely from peritoneal dialysis infections. It is intrinsically resistant to Vancomycin but sensitive to ampicillin. So, diagnosis of this bacteria is important if isolated from PD infections.

**Case presentation.:**

An elderly female patient of ESRD on continuous ambulatory peritoneal dialysis (CAPD) was admitted with complaints of high fever and cloudy PD effluent for 2 days. She was started with vancomycin and imipenem empirically but did not improve even after 4 days. Pus cells were seen when PD fluid was examined microscopically. BACTEC culture of PD fluid isolated growth of Gram-positive cocci, which was confirmed as *Pediococcus pentosaceus *. It was resistant to vancomycin. The antibiotic of the patient was changed to ciprofloxacin IV. The patient responded in 2 days and was discharged after 7 days.

**Conclusion:**

This is the first case report of *Pediococcus pentosaceus* peritonitis in an ESRD patient on CAPD. Accurate diagnosis and antibiotic sensitivity test of the bacteria is important especially if isolated in critical patients as it is intrinsically resistant to vancomycin.

## Introduction

Peritoneal dialysis (PD) is one of the therapeutic options for end-stage renal disease (ESRD). Worldwide, about one-tenth of ESRD patients are on PD [1]. Peritonitis is one of the common complications of PD. It has been associated with morbidity and mortality. Peritonitis is a major cause of PD failure and change of therapy to haemodialysis [2–4]. In about 50% of cases, peritonitis is caused by Gram-positive bacteria; in 15–20% , by Gram-negatives; and in about 4% by a mixture of micro-organisms [5, 6]. Various new and rare micro-organisms like *Lactobacillus acidophilus *, *Leuconostoc* spp, *Rhodococcus equi , Rhizobium radiobacter , Moraxella Osleonsis, Kingella Denitrificans* etc*.* has also been reported to cause peritonitis from different places [7–12].

Previously Pediococcus , an emerging pathogen, has been reported to cause bacteraemia, liver abscess, etc. [13–15]. Pediococcus is a Gram-positive cocci arranged in pairs, tetrads or clusters [16]. It has been isolated from stools of normal humans, patients on antibiotics for gut decontamination, blood cultures from septicaemia cases, abdominal abscess. Its pathogenic role has not been established in all cases. In those cases where it was implicated, *Pediococcus acidilactici* was the causative agent. *Pediococcus* *pentosaceus* has been reported from very few cases [13, 16]. Here we are reporting a case of peritonitis caused by *Pediococcus pentosaceus* in a patient undergoing PD.

## Case Report

A 71-year-old female patient of ESRD was started on continuous ambulatory peritoneal dialysis (CAPD). After 2 months, she was admitted to the Nephrology ward with complaints of high fever and cloudy PD effluent for 2 days. She had a history of type 2 diabetes mellitus (DM) for the last 15 years. On examination she had a fever of 103° F and pain on the abdomen. PD effluent was purulent. Other systemic examinations were normal. On ultrasonography, no abdominal fluid collection was seen. She was started with vancomycin and imipenem empirically but did not improve even after 4 days.

PD fluid was sent for microbiological analysis. White blood cell (WBC) count of the fluid was 960 µl^−1^. Microscopy of the fluid showed pus cells but no bacteria were seen. Sample was cultured in BACTEC 9120 (BD Biosciences, USA). Culture was positive after 48 h of aerobic incubation. BACTEC subculture was done on blood agar and MacConkey agar. On blood agar, small non-haemolytic colonies were seen whereas small lactose-fermenting colonies grew on MacConkey agar. The isolated organism was non-motile, non-spore forming, Gram-positive cocci arranged in tetrads. The bacteria grew in the presence of 6.5% NaCl and was catalase negative, oxidase negative and pyrronidonyl aryl amidase (PYR) test negative. It was identified as *Pediococcus pentosaceus* in Phoenix automated system (BD Biosciences, USA). The identity was also confirmed by Vitek-MS (bioMerieux, France). The antibiotic sensitivity by the same system showed resistance towards vancomycin but the isolate was sensitive to ampicillin, amikacin, ciprofloxacin and doxycycline. Following the microbiological diagnosis, Ciprofloxacin 400 mg IV twice a day was given to the patient for 7 days. The PD catheter was removed and the patient was put on hemodialysis. After 3 days, the fever and abdominal pain subsided. Subsequent peritoneal fluid culture was sterile. The patient was discharged in a stable condition with advice to continue oral ciprofloxacin 500 mg twice daily for a further 7 days.

## Discussion

*Pediococcus* spp. is a Gram-positive, catalase and oxidase-negative cocci that may be either α-haemolytic or non-haemolytic [17]. Because of their reaction with group D streptococcal anti-sera, they may be overlooked and erroneously identified as streptococci. Suspicion should be raised , however, by their susceptibility to penicillin and ampicillin (and generally aminoglycosides) and resistance to vancomycin [18–21]. Originally thought to be clinically insignificant, pediococci have been associated with human infection since 1987 [18]. *Pediococcus* spp. is found on fermenting vegetables and in silage, dairy products and beer. The bacteria have been used in the processing and preservation of selected meats, vegetables, cheese and soy products. Human isolates have been obtained from saliva, stool, urine, sputum, wounds, abscesses and blood [13, 22, 23]. *Pediococcus pentosaceus* has never been found to be associated with peritonitis till now.

Peritonitis is a cause of mortality in PD patients contributing to about 16% of deaths of patients on CAPD [24]. Though its incidence has been decreased due to improved techniques and better devices, it is still a major cause for PD failure [1, 25, 26]. Infection with rare, drug-resistant bacteria like *Pediococcus spp*. can further add to the diagnostic dilemma and treatment failure. Our isolate was identified by conventional methods like Gram stain and biochemical tests and confirmed by Phoenix automated system (BD Biosciences, USA) and Vitek -MS (bioMerieux, France).

*P. pentosaceus ,* as a pathogen, has been given attention recently due to improved diagnostic methods [27]. *P. pentosaceus* is an opportunistic pathogen and can infect a person debilitated by a major underlying disease [28]. An update onperitoneal dialysis-related infections has recommended the use of a first generation cephalosporin or vancomycin as an empirical therapy for Gram-positive organisms [29]. Though our isolate was resistant to vancomycin it was sensitive to ampicillin. Selection of the higher antibiotic empirically would have had no benefit in our patient, and herein lays the clinical relevance of this case. Inherent vancomycin resistance of pediococci may help these bacteria to persist in the hospital environment, but their potential to cause nosocomial infections is yet to be evaluated. These vancomycin-resistant bacteria, like *Leuconostoc* spp. and *Weissella* spp., are becoming clinically important as opportunistic human pathogens. In the bacteriology laboratory, they can be reported as unidentified Gram-positive cocci or can be misidentified as variants of enterococci, a more common human pathogen [16]. This results in under-reporting of these rare pathogens and can also lead to erroneous diagnosis and inappropriate management.

### Conclusion

This is the first case report of *Pediococcus pentosaceus* peritonitis in an ESRD patient on CAPD. Careful interpretation of biochemical tests and sensitivity pattern is required to identify these rare isolates and for proper treatment. Possibility of such opportunistic agents should be considered in the case of immunocompromised and debilitated patients. Further studies are needed to ascertain the pathogenicity of such organisms.
